# A New Trephine

**Published:** 1860-10

**Authors:** 


*A New Trephine* has been invented by Dr. Galt, of Va. It differs from the old trephine in being conical and having the crown teeth oblique, the teeth divided by grooves extending up the outside of the cone, so as to form cutting ribs something after the fashion of a screw. It proposes to cut only through the cranial plates, when from its cone-shaped ribs, it ceases to cut by becoming wedged. B.

